# Natural Products in Anticancer Activity: 2nd Edition—From Chemical Diversity to Mechanistic and Translational Insights

**DOI:** 10.3390/molecules31213823

**Published:** 2026-10-23

**Authors:** Luciana Scotti, Marcus Tullius Scotti

**Affiliations:** Cheminformatics Laboratory—Postgraduate Program in Natural Products and Synthetic Bioactive, Federal University of Paraíba, João Pessoa 58051-900, PB, Brazil; mtscotti@gmail.com

## 1. Introduction

Natural products continue to occupy a distinctive position in anticancer research, not only as sources of structurally diverse molecules but also as starting points for uncovering biological mechanisms and developing innovative therapeutic strategies. Their chemical complexity provides access to molecular scaffolds with a broad range of biological properties, while their interactions with interconnected cellular pathways can reveal new opportunities for intervention in cancer.

The Special Issue “Natural Products in Anticancer Activity: 2nd Edition” brings together seven contributions that collectively illustrate this expanding research landscape. The studies encompass marine-derived peptides, lignin, a semisynthetic thymoquinone derivative, curcumin, a plant extract, a nanostructured delivery system containing quercetin and rosmarinic acid, and newly identified diterpenes from *Jatropha ribifolia* [1,2,3,4,5,6,7]. Despite their considerable chemical and methodological diversity, these contributions share a common objective: to investigate how naturally derived or natural-product-inspired substances can interfere with processes associated with tumor development and progression.

Importantly, the studies also illustrate the increasing integration of chemical, biological, computational, and pharmacological approaches. Rather than focusing exclusively on the identification of cytotoxic effects, the contributions address molecular mechanisms, signaling pathways, transcriptomic responses, immune modulation, toxicological aspects, and drug-delivery strategies. Viewed together, they provide a representative snapshot of the multiple dimensions currently shaping natural-product anticancer research.

## 2. Chemical Diversity and the Discovery of Anticancer Candidates

The chemical diversity represented in this Special Issue begins with the diversity of biological sources explored.

Zhang et al. [1] investigated the histidine-rich peptide rAj-HRP from the sea cucumber *Apostichopus japonicus*. In HCT116 colon cancer cells, rAj-HRP inhibited proliferation, migration, and adhesion to the extracellular matrix and affected the cellular cytoskeleton. These effects were associated with apoptosis and modulation of EGFR-associated signaling, including AKT, PLCγ, p38 MAPK, and c-Jun, together with reduced MMP2 expression. The in vivo experiments further demonstrated reductions in tumor volume and weight without an associated effect on body weight [1]. This study highlights the potential of marine-derived peptides as biologically active molecules capable of influencing several processes involved in tumor progression.

A markedly different natural material was investigated by Kashimoto et al. [2], who characterized low-molecular-weight lignin obtained from Japanese cedar and *Eucalyptus globulus* by microwave-assisted acid-catalyzed solvolysis. The resulting lignin preparations affected tumor-cell viability through modulation of apoptotic pathways, including increased TNF-α, Fas, and FasL and suppression of NF-κB and mTOR. In tumor-bearing mice, repeated treatment inhibited tumor growth and was associated with increased caspase-3 activity [2]. At the same time, the authors highlighted the still incompletely understood relationship between lignin structure and antitumor activity, an issue of particular importance for complex natural materials.

The discovery of new chemical entities is represented by the work of Souza et al. [7], which reports eight previously undescribed macrocyclic diterpenes, ribifolones A–H, from *Jatropha ribifolia*. Among the compounds evaluated against SK-MEL-28 melanoma and HCT116 colorectal cancer cells, jatrophone showed the strongest cytotoxic activity, whereas ribifolone C displayed moderate activity [7]. Beyond the identification of new structures, the study demonstrates the value of exploring under-investigated botanical sources, particularly those associated with biodiverse regions such as the Brazilian semi-arid region.

Together, these studies reinforce a fundamental premise of natural-product research: chemical diversity remains an important starting point for discovering molecules and molecular frameworks with potential biological relevance in cancer.

## 3. From Anticancer Activity to Mechanistic Insights

The identification of biological activity represents only the beginning of the process of understanding a potential anticancer agent. Several contributions to this Special Issue move beyond phenotypic observations to investigate cellular and molecular responses.

In addition to demonstrating the antitumor effects of rAj-HRP, Zhang et al. [1] investigated intracellular signaling and molecular markers associated with apoptosis, providing evidence connecting the observed cellular effects with EGFR-related signaling and apoptotic pathways.

El-Far et al. [3] examined TQFL19, a newly synthesized derivative of thymoquinone, in triple-negative breast cancer models. TQFL19 displayed greater cytotoxic activity than thymoquinone against the evaluated cancer cell lines and induced apoptosis and cell-cycle arrest in selected models. In vivo, the compound inhibited tumor growth, migration, and metastasis in a 4T1 allograft model [3]. The study illustrates how modification of a natural-product scaffold can generate derivatives with distinct biological properties and potentially broaden the chemical space available for anticancer research.

Curcumin was investigated through a complementary systems-level strategy by Mashozhera et al. [4]. In U-87 MG glioblastoma cells, curcumin reduced cell viability and proliferation and induced apoptosis. Global gene-expression profiling identified 5036 differentially expressed genes and 13 dysregulated cancer-associated pathways, including MAPK, Ras, TGF-β, Wnt, cytokine, and TNF-related signaling [4]. These findings illustrate how transcriptomic approaches can expand mechanistic investigations by revealing coordinated molecular responses rather than focusing on individual targets.

Taken together, these studies demonstrate the value of connecting observable cellular phenotypes with molecular evidence. Such integration is particularly relevant in cancer research, where biological responses frequently involve interconnected pathways rather than a single molecular event.

## 4. Integrating Omics, Network Pharmacology, and Molecular Modeling

The increasing incorporation of computational and systems-level approaches represents another important feature of the contributions.

The transcriptomic analysis of curcumin [4] provides a broad molecular view of cellular responses, whereas the study of J. ribifolia [7] combines network pharmacology, molecular docking, and molecular dynamics with experimental cytotoxicity data. Network pharmacology suggested the involvement of the PI3K-AKT-mTOR axis, while molecular modeling was used to investigate interactions between selected compounds and proteins [7].

These approaches can substantially expand the capacity to generate and prioritize mechanistic hypotheses. At the same time, their interpretation requires an important distinction between predicted molecular interactions and experimentally validated targets. Computational approaches are particularly valuable when used in conjunction with biological evidence, providing a rational framework for selecting hypotheses for subsequent experimental testing.

The integration of omics and computational methodologies with experimental pharmacology therefore represents an important direction for the field. Rather than replacing experimental investigation, these approaches can help organize complex biological information and guide the design of more focused validation studies.

## 5. Safety, Immunomodulation, and Drug Delivery

The therapeutic potential of an anticancer agent depends not only on its ability to affect tumor cells but also on its safety, selectivity, pharmacological behavior, and capacity to reach the relevant biological compartment.

Costa et al. [5] addressed several of these aspects in their investigation of a saline extract from *Euphorbia hirta* leaves. The authors combined phytochemical characterization with acute toxicity, hematological and biochemical analyses, histopathology, genotoxicity assessment, and antitumor experiments using the sarcoma 180 model. The extract demonstrated antitumor activity in vitro and in vivo, with reductions in tumor weight of approximately 70% at the evaluated doses. Under the experimental conditions investigated, no mortality, major organ damage, or genotoxic effects were observed. The study also evaluated several cytokines, providing evidence of immunomodulatory responses associated with treatment [5].

A different translational challenge was addressed by Stoyanova et al. [6]. Quercetin and rosmarinic acid possess biological potential but are limited by physicochemical properties including low water solubility, stability, and bioavailability. The authors developed PLA/PEG composite nanofibers for the co-delivery of both compounds, achieving high entrapment efficiency and a biphasic release profile. In vitro evaluation using SH-4 melanoma cells and HaCaT keratinocytes demonstrated anticancer activity together with lower toxicity toward the normal-cell model [6].

These studies emphasize that natural-product research must increasingly consider the conditions required for biological activity to become therapeutically meaningful. Toxicological evaluation, selectivity, formulation, controlled release, and bioavailability are not secondary considerations but important components of the development pathway.

## 6. Emerging Perspectives from the Special Issue

Taken together, the contributions suggest that the future of natural-product anticancer research will depend increasingly on integration rather than isolated methodological advances.

Chemical characterization needs to remain connected to biological phenotypes; mechanistic hypotheses need to be tested experimentally; computational predictions need to be interpreted alongside molecular and cellular evidence; and biological efficacy needs to be considered together with safety, selectivity, pharmacokinetics, and delivery.

This integrated perspective is particularly relevant for natural products because their biological effects may emerge from interactions involving multiple molecular pathways and biological processes. The studies included in this Special Issue illustrate different points along this continuum, from the discovery of new chemical entities to the investigation of signaling pathways, transcriptomic responses, immune modulation, toxicological profiles, and formulation strategies [1,2,3,4,5,6,7].

Several challenges nevertheless remain. For complex extracts and materials, reproducible chemical characterization and standardization are essential. For isolated compounds and derivatives, identification and experimental validation of molecular targets remain important steps. Computational and systems-level approaches offer valuable opportunities for generating hypotheses, but their predictions require appropriate experimental confirmation. Finally, advances in formulation and delivery will be increasingly important for overcoming limitations related to solubility, stability, bioavailability, and pharmacokinetic behavior.

In this context, multidisciplinary integration may represent one of the most productive directions for the field. The combination of natural-product chemistry, molecular pharmacology, omics, computational science, toxicology, formulation, and experimental oncology can provide a more comprehensive framework for evaluating the therapeutic potential of natural products.

## 7. Concluding Remarks

The second edition of “Natural Products in Anticancer Activity” reflects not only the continuing richness of nature as a source of chemical diversity but also the increasing sophistication of the approaches used to investigate it. The seven contributions span different biological sources, molecular classes, cancer models, experimental strategies, and stages of preclinical development, illustrating the breadth of contemporary research in this area [1,2,3,4,5,6,7].

More importantly, the collection reflects a progressive movement from the identification of biological activity toward an integrated understanding of chemical structure, molecular mechanisms, tumor biology, safety, and therapeutic delivery. Discovery, mechanism, computation, toxicology, and formulation are increasingly interconnected components of the same research process.

At the same time, the studies remind us that promising experimental activity is a starting point rather than an endpoint in anticancer drug discovery. Target validation, selectivity, reproducibility, standardization, pharmacokinetics, bioavailability, and appropriate experimental models remain essential considerations for future development.

We hope that the contributions presented in this Special Issue will stimulate further multidisciplinary investigations and strengthen the connection between natural-product chemistry, mechanistic pharmacology, computational science, and translational oncology. Ultimately, continued integration of these disciplines may help transform promising natural products and natural-product-inspired molecules into experimentally validated and pharmacologically meaningful anticancer candidates.

## Data Availability

No new data were created or analyzed in this study. Data sharing is not applicable to this article.
